# Digital resources as an aspect of teacher professional digital competence: One term, different definitions – a systematic review

**DOI:** 10.1007/s10639-022-11321-z

**Published:** 2022-09-30

**Authors:** Sandra Heine, Matthias Krepf, Johannes König

**Affiliations:** Empirical School Research, University of Cologne, Gronewaldstr. 2, 50931 Cologne, Germany

**Keywords:** Digital resources, Definition, Teacher competence, Conceptual construct

## Abstract

The term ‘digital resources’ is increasingly used in educational research to describe the specific knowledge and skills that constitute teachers’ professional digital competence. Educational policy documents, including the European Framework for the Digital Competence of Educators (DigCompEdu), deploy the term to reaffirm teachers’ need for special skills in using digital resources. However, educational research literature presents inconsistent views of the term, restricting its effective use in further research and the promotion of associated skills among pre-service and in-service teachers. To clarify the term ‘digital resources’ and support future research related to its application especially in empirical research on teachers’ professional digital competence, this systematic review aims to analyse the definitions of digital resources as a scientific term in 23 articles and to examine and compare the facets and aspects of digital resources. Finally, we derive a definition from the various perspectives and discuss the implications for the definition of digital resources as an aspect of teachers’ professional digital competence.

## Introduction

As the digitalisation of society progresses, its influence on the education sector increases (Bottino, 2020). Students must prepare for this digital world by acquiring the appropriate competences (Willis et al., 2019). This is a significant responsibility for their (prospective) teachers, who must mediate and promote the students’ competences. Demands on teachers have consequently not only changed but also increased (Mußmann et al., 2021). Teachers’ role needs a redefinition (Bottino, 2020). The challenges associated with the recent COVID-19 pandemic in particular have highlighted these concerns worldwide (Yazgan, 2022).

To meet increased demands, relevant content must be incorporated into teacher training and further education. This requires the systematic clarification of key terms and concepts (Spante et al., 2018), including the term ‘digital resource’. It frequently appears in the literature, has been deployed in various ways and has its own area of competence in educational policy documents, such as the European Framework for the Digital Competence of Educators (DigCompEdu) (Redecker, 2017). However, a first screening of the literature reveals that very different aspects are addressed partially with the same term. As Kempe and Grönlund (2019) summarised, ‘The terminology used in research literature when addressing digital learning resources is not consistent or well defined’. To the best of our knowledge, the term has not been systematically addressed since.

Consistent understanding is crucial for teacher training and further education so that teachers' necessary competencies in this area can be defined and promoted in a targeted manner. Empirical approaches to corresponding measuring and testing must also be able to draw on a consistent definition of the construct.

Against this background, we performed a systematic literature search using the term ‘digital resources’ in relation to teachers and their professional digital competence. The final corpus for this review includes 23 articles that present definitions for the conceptual construct of ‘digital resources’ in current educational research. To systematically capture the different aspects and facets, we have coded the articles according to their use of the conceptual construct. We subsequently converted the results of the coding into a generally valid definition. This definition should be used to define the area in which teachers’ professional digital competence in dealing with digital resources should be promoted in the future.

## Research status – An overview

Current educational research presents several different views on digitalisation. It is impossible to provide an in-depth description of the current state of research in all its diversity in a single article. Therefore, we focus on two areas that affect this review: (prospective) teachers’ professional digital competence and the digital resources themselves.

### (Prospective) teachers’ professional digital competence

Educational research has been concerned with the teachers’ competence in pedagogical settings for decades. Teacher competence may be defined as ‘context-specific, cognitive performance dispositions that are functionally responsive to situations and demands in certain domains’ (Kaiser & König, 2019). As generic models of professional competence demonstrate, cognitive and affective-motivational areas are both included (e.g., Blömeke, 2017), in accordance with Weinert’s (2001) concept of competence. The cognitive aspects of teacher competence have been a particular focal point, following Shulman (1987) who introduced a classification of teacher knowledge that researchers typically use to differentiate between teachers’ content knowledge (CK), pedagogical content knowledge (PCK), and general pedagogical knowledge (GPK) (Guerriero, 2017). This range of professional knowledge is crucial to teachers’ mastery of the core challenges of teaching (Shulman, 1987) and therefore contributes to teacher professional competence. However, teacher competence can be considered to transcend knowledge (Shavelson, 2010). An important extension has been described by Blömeke et al. (2015), highlighting situation-specific skills that mediate between cognitive dispositions (e.g., teacher knowledge) and teacher performance in the classroom. Situation-specific skills are regarded as more proximal to teacher performance than teacher knowledge (König et al., 2021a). Several approaches have adopted this modified focus on teacher competence. Current research on teacher competence is challenged to relate to and expand its frameworks and models to the new professional demands that teachers must meet in the transformative process of digitalisation at school (McFarlane, 2019; Selwyn, 2012).

Originating from another direction, educational research concerning the integration of technology into classroom teaching has also produced models and frameworks relating to teachers’ professional digital competence. For example, in Puentedura's SAMR—substitution, augmentation, modification, redefinition—model (2014), the focus is on the idea of innovation. The model encourages teachers to achieve a higher level of teaching and learning processes through more innovative use of technology in the classroom (Hamilton et al., 2016). Koehler and Mishra (2009) described that this is accompanied by new challenges for teachers. They underlined their statement by expressing the complex interplay of pedagogical, technological and content-related knowledge with the technological pedagogical content knowledge (TPACK) model (Mishra & Koehler, 2006). This model extends Shulman’s model aforementioned by adding ‘technological knowledge’ (TK). The various intersections between TK, pedagogical knowledge (PK) and CK are often represented using a Venn diagram (Mishra & Koehler, 2006). TPACK itself is the resulting intersection of all knowledge categories of the teachers, the so-called ‘technological pedagogical content knowledge’. In addition to TK, PK and CK, these include TPK (technological pedagogical knowledge), TCK (technological content knowledge) and PCK. However, Mishra and Koehler emphasise that an isolated approach to TK in teacher training and further education is insufficient and that both CK and PK must be considered consistently (Mishra & Koehler, 2006). The extent to which this also applies to the digital resources will only become apparent when their placement in the TPACK model can be conceptually clarified. In this context, it is important to note that the model originally considered both analogue and digital forms with the term ‘technology’ (Koehler & Mishra, 2009). Meanwhile, the model is cited almost exclusively in the digital context, as a literature review by Setiawan et al. (2019) reveals. TPACK has also been used as a basis for the creation of other models (Brevik et al., 2019).

The TPACK model reveals that digitalisation is not simply about developing new knowledge facets but also about linking them to existing ones. This makes integration into the education sector even more difficult (Brantley-Dias & Ertmer, 2013)—a fact that the SAMR model considers only to a limited extent. With the focus on innovation, the product is prioritised over the process (Hamilton et al., 2016). This indicates that teaching and learning processes can be improved by simply using digital technology. The SAMR model thereby disregards situational requirements, which are represented by the P (pedagogical) in the TPACK model and considered in Blömeke et al.’s (2015) competence model in relation to situation-specific skills. In light of teaching’s complexity, it is impossible to record and evaluate all situation-specific skills in a single study.

However, it is possible to approach the complexity of the different situations in a single lesson. When developing lesson plans, teachers must anticipate different situations and identify appropriate courses of action. Thus, an evaluation of the situation-specific skills is possible. König et al. (2021b) choose this approach. Using the CODE-PLAN model (*cognitive demands of lesson planning*), teachers’ planning competence can be described and analysed using lesson plans. In this way, different situations and the skills associated with them can be recorded. Various models also take the integration of information and communications technology (ICT) into account, such as the Technology Integration Planning Cycle (Hutchison & Woodward, 2014) or the Will, Skill, Tool Model of Technology Integration (Knezek et al., 2003). In addition to the description, the evaluation of ICT integration in lesson plans is key. In their literature review, König et al. (under review) summarise the results of studies that deal with, among other things, teachers’ ability to describe and justify the integration of ICT in the classroom. The conceptual link between planning and decision-making as a competence helps here.

### Digital resources as defined in the DigCompEdu

The examples outlined hitherto reveal that teachers’ competences are crucial for successful digitalisation in schools. Various policy documents address this fact. In Germany, for example, teachers’ competences in dealing with ‘digital educational media’ are already described in the strategy paper ‘Bildung in der digitalen Welt’ of the conference of Ministers of Education and Cultural Affairs in 2016. One year later, the European Commission published DigCompEdu. As a result of the analysis and comparison of various national and international frameworks, the report provides common guidelines for European states. It can thus be regarded as a means of understanding between countries. The framework integrates six distinct competence areas.

Competence area 6 shows that, to a considerable extent, teachers are responsible for the mediation and promotion of learners’ so-called ‘key competencies’. This occurs not only explicitly but also implicitly through the teachers’ characteristics as role models. Their approaches to digital resources also shape students’ learning processes. During the COVID-19 pandemic, in particular, digital resources were essential in facilitating distance learning from home for students (Mußmann et al., 2021). To continue teaching online, teachers had to acquire new competences relating to digital resources or expand existing competences. According to a study entitled ‘Digitalisierung im Schulsystem’, 70% of teachers in Germany created digital resources for their lessons themselves. A further 22% responded that they had partly created digital resources (Mußmann et al., 2021). However, creation is not the only thing in which teachers need to develop or enhance their competencies related to digital resources as the following insight in this area shows.

According to DigCompEdu, teachers require the following competencies with respect to digital resources (Redecker, 2017):

- Selecting digital resources
- Creating and modifying digital resources
- Managing, protecting and sharing digital resources

The individual components are subsequently explained in greater detail (Redecker, 2017). In this context, the close connection to the open educational resources (OER) becomes apparent followed by the development and promotion of the 4Cs (creativity, critical thinking, communication, collaboration). A focus on the latter ‘is essential to prepare students for the future’ (Bedir, 2019). Given these networks and the wide range of skills associated with digital resources, the significance of this area of competence crystallises. Thus, the definition of the term becomes more important.

DigCompEdu defines the term ‘digital resource’ as follows (Redecker, 2017):

“The term usually refers to any content published in computer-readable format. For the purposes of DigCompEdu, a distinction is made between digital resources and data. Digital resources in this respect comprise any kind of digital content that is immediately understandable to a human user, whereas data need to be analysed, treated and/or interpreted to be of use for educators.”

In addition to data, hardware is excluded from this definition. Hence, according to DigCompEdu, laptops, smartphones, tablets and interactive whiteboards are not considered digital resources (Redecker, 2017).

Current research in the field of education indicates that there are different views on this. Thus, no common understanding of digital resources exists, and that presented in DigCompEdu has not been adopted by researchers in the field: only two articles in the present review refer directly to it (Maiier & Koval, 2021; Štemberger & Konrad, 2021), while another simply mentions the framework (Rodríguez-Muñiz et al., 2021). The term ‘digital resources’ encompasses a broad scope, ranging from apps for smartphones and tablets (Nilsen et al., 2020) to computers and servers (Ukah, 2020). In an initial overview, it therefore includes both software and hardware. However, this lack of consistency in the definition not only affects research (Kempe & Grönlund, 2019) but also leads to confusion regarding terminology among users, as Alberola-Mulet et al. (2021) perceive: ‘Finally, it is worth noting that the teachers in the sample generally confused the terms “digital resources” and “new technologies”’ (p. 11). However, if the term itself is not clearly defined, how can the associated skills be promoted in a targeted manner?

## Research questions

To clarify the term ‘digital resources’, our review pursues the goal outlined below and addresses the following research questions (RQ).

- **RQ 1:** What definitions exist for the conceptual construct ‘digital resources’ in current educational research?
- **RQ 2:** What aspects and facets are included into the conceptual construct ‘digital resources’ in thefield of educational research?
- **RQ 3:** Where can digital resources be conceptually placed in the TPACK model?
- Aim: What general definition and conceptual understanding can be derived from the different perspectives in current educational research for the term ‘digital resources’?

## Methods

To provide an overview of the existing definitions of the conceptual construct ‘digital resources’, we decided to prepare a systematic review. The approach of a systematic scientific literature review can provide comprehensive insights into the current state of educational research on the topic of digital resources. This makes the definitions used and the conceptual understanding explicit. For a clear presentation we followed the "PRISMA 2020 explanation and elaboration" by Page et al. (2021).

### Systematic literature search

A systematic search for suitable literature was conducted in December 2021 using the literature databases Web of Science (WoS) and Education Resources Information Center (ERIC). In WoS, the query encompassed *title*, *abstract* and *author keywords* using the search terms *digital* + resource* AND teach*. The ‘ + ’ ensured that this specific term was searched for. A search query without ‘ + ’ delivered significantly more results. However, searches for the two terms separately revealed they often were detached from one another and therefore not expedient for the review. On the one hand, teach* restricted the results to the educational context. On the other hand, it ensured that all different forms of the term (e.g., ‘teacher’, ‘teaching’, etc.) were considered. This yielded 396 results.

**Fig. 1:**
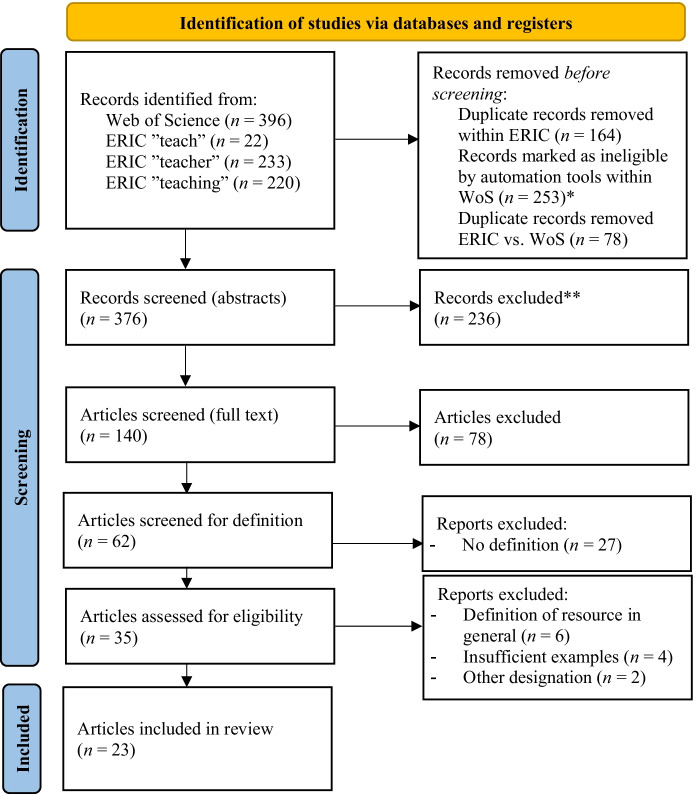
Flow diagram (following the guidelines of Page et al., 2021) * Used automation tools: Document Types ➔ Articles; Languages ➔ English ** Records were excluded after coding the abstracts

The query using ERIC was similarly implemented. Since ERIC includes no option for automatic truncation, the variation was implemented manually. Three different enquiries were made in total using the following forms: *teach*, *teacher* and *teaching*, each in combination with *digital* + *resource*. The change from singular to plural did not affect the number of hits. Figure 1 presents the results of the different enquiries.

### Inclusion and Exclusion criteria

Using the automation tools, the results were then filtered by language (English) and form (articles). Only peer-reviewed articles returned in ERIC were considered to ensure the quality of the publications*.* Finally, all duplicates were removed. This resulted in a provisional corpus of 376 articles whose records, including abstracts, were exported to Excel.

All abstracts were then coded to better estimate relevance. The coding included whether the study was empirical, which group of people was examined, which school type was the focus, whether a single resource was considered, whether a specific school subject was involved and which country the article concerned. This coding aimed to provide an overview of the state of research on the subject of ‘digital resources’ and to exclude articles that focused on single resources, addressed digital resources in early childhood education and did not deal teachers’ use of them. This did not apply to articles that clearly indicated in the abstract that the focus was on not only digital resources as an object but also as a concept. In this way, 236 articles were excluded (Table 1).

**Table 1:** Exclusion and inclusion criteria (abstracts)

Exclusion criteria	Inclusion criteria
- single resource in focus - no (school) educational context - early childhood education - use by students only - strong focus on subject-specific use	- use and deployment of digital resources by (prospective) teachers - general discussion of digital resources in the school educational context - transferability of possible subject-specific results

Next, the 140 remaining article PDFs were subjected to a full-text screening to investigate for the use of the term ‘digital resource’ using the automatic search function. The search was flexibly adjusted: for example, if acronyms were used in the abstract, the full text was also scanned for them. Some texts were searched only for the use of the term ‘resource’ to identify insertions such as ‘learning’. This made it possible to determine whether the articles only used the concept generally. If no attempt to explain the term was discernible, the articles were excluded. This affected a further 78 articles.

The 62 remaining articles were carefully assessed for eligibility. First, the articles were examined to determine whether they included concrete definitions or examples that would allow conclusions to be drawn from the authors’ understanding of the term, and 27 articles did not meet the criteria. Three representative examples are given below to illustrate the exclusion process transparently and comprehensibly. For example, Klucevsek and Brungard (2020) refer to literature databases and management programmes as digital resources but do not extend this into a general context. Instead, the term is later used for enumeration without further explanation: ‘Research shows that many students worry about this prospect but accept it as “part of the deal” if they want the convenience of websites, apps, and digital resources’ (p. 612). Therefore, the authors’ understanding of the terms cannot be deduced. Araujo et al. (2017) also give examples with ‘an ever-expanding array of digital curriculum resources (e.g., tasks, videos)’ (p. 687) but do not specify any further. The term ‘resource’ later becomes a sort of representative: ‘Because of the dominance of video in flipped classes we often refer to these resources as “video” rather than “video and multimedia”’ (p. 688). The term thus remains vague in its usage and does not enhance our understanding. Clarification of the term is even more difficult if, in addition to the lack of a definition, digital and non-digital resources are poorly distinguished, as with Nussbaum and Diaz (2013). In this case, both digital and analogue content and activities are listed as examples of resources.

Finally, passages in the remaining 35 articles that were previously identified as candidates for a definition or as ‘conducive to understanding the term’ were examined in detail. The analysis revealed that the definitions in six articles referred only generally to ‘resources’ and were not limited to digital (Gueudet & Trouche, 2009; Kynigos et al., 2020; Pattanshetti et al., 2018; Pepin et al., 2017a, b; Svendsen & Svendsen, 2021; Tapan-Broutin & Ilkorucu, 2021). Two more recent articles by Pepin, Gueudet and Trouche are included in the final corpus of this review, and so their understanding of the term is considered. Four further articles were excluded because they included too few examples and it was thus impossible to narrow down the term. As a ‘cut-off criterion’ it was finally determined that at least three examples should be provided to facilitate differentiation. Neither was this the case with Mkhize and Davids (2021): ‘Digital resources refer to hard- and software resources and connectivity to the internet’ (p. 22). While the terms hardware and software refer more to categories, they are comprehensive in combination and without further specification or explanation. Hence, no differentiation is possible for the term ‘digital resource’. This applied also to the articles by Hashey and Stahl (2014), Nilsen et al. (2020) and Kemp and Jones (2007). Two other articles used a different designation: Karlsudd’s (2018) definition referred to the digital resources as ‘learning aids’, and the associated explanation also had this focus. However, the other designation permits no conclusions regarding the understanding of the term, which we attempt to clarify in this review. Ruiz-Cabezas et al. (2020) provide several examples, but these are listed under ‘ICT resources’. In this case, it was unclear whether this was intended to emphasise a different focus. Owing to these uncertainties, the articles were excluded (Table 2).

**Table 2:** Exclusion and inclusion criteria (full texts)

Exclusion criteria	Inclusion criteria
- no definition of terms recognisable - specification only by naming a single example - vmixing of different terms - conceptual construct no longer appears in continuous text	- many examples that allow a general understanding and a delimitation of the term - clear distinction from other terms - exact explanation of the understanding of the term - consistent use of the term

The exclusion and inclusion criteria outlined above yielded a final corpus of 23 articles, which were coded to depict the various aspects and facets of the conceptual construct ‘digital resources’.

### Basic characteristics of articles

**Fig. 2:**
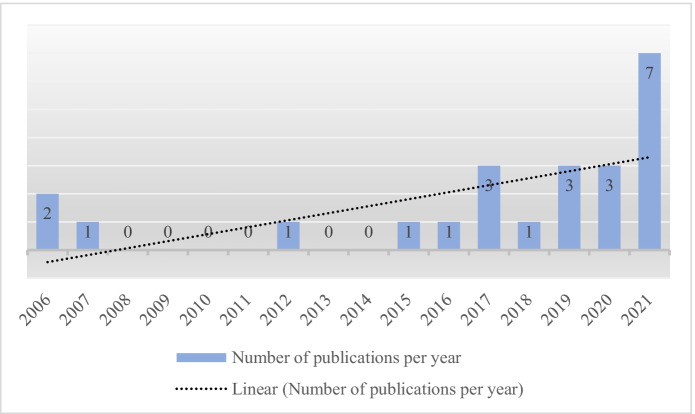
Trend of publications concerning digital resources

Figure 2 represents the increased interest in educational research in the field of digital resources. This figure relates only to our 23 selected publications and is not universally valid but merely serves to visualise the trend.

The COVID-19 pandemic’s impact on research in the field of digitalisation in teacher education cannot be ignored (Carrillo & Flores, 2020). The beginning of the pandemic in 2019 saw an increase in publications compared to the previous year. Even in 2020, as the virus spread and emergency remote teaching was widely implemented, the number remained unchanged. In 2021, there was a provisional high of seven publications within the year.

### Development of coding schemes and coding

This section describes the development of coding schemes. The coding aimed to capture the defining facets and aspects in a standardised way. In addition, we looked at the defining parts of the articles in a whole to classify the existing definitions and to give an overview of the conceptual construct ‘digital resource’ in educational research. The procedure for this is depicted in a later section.

#### Aspects and facets of digital resources

**Fig. 3:**
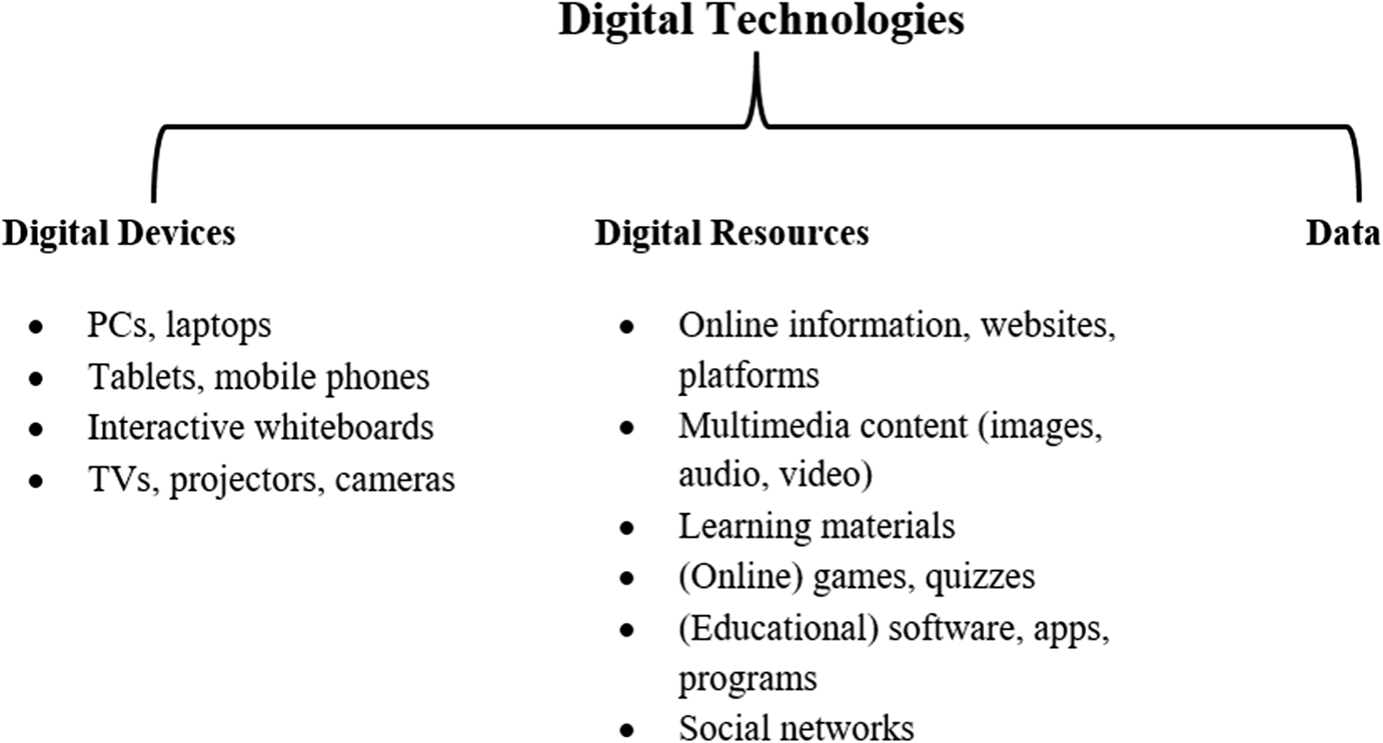
Key concepts used in DigCompEdu

Initial coding comprised the structural distinction between ‘hardware’, ‘digital resource’ and "data", following the categorisation in DigCompEdu (Redecker, 2017), followed by a further subdivision of the ‘digital resource’ category by means of the examples listed in DigCompEdu (see Fig. 3). Preliminary examination of the selected articles led to the addition of the subcategories ‘emails’, ‘e-books/digital textbooks’, ‘blog’, ‘simulation’ and ‘cloud’. Moreover, beyond creating categories, several articles provided concrete examples. These were then classified as representative of the respective (sub)category during coding.

Although some articles include the conceptual construct ‘digital resource’ in their title, it is not only in these that other terms are used synonymously. This applies to both the partial term ‘digital’ and the term ‘resource’. To register this systematically, the following synonyms were included for the partial term ‘digital’: ‘multimedia’, ‘technological’, ‘ICT’, ‘electronic’ and ‘online’. The synonyms ‘device’, ‘tool’, ‘technology’, ‘ICT’ and ‘artefact’ were listed for the partial term ‘resource’. For further detail, see the developed coding schemes in the Appendix Table 4.

Given that some articles emphasise the educational context using correspondent insertions or additions, such as ‘curriculum’, ‘educational’, ‘instructional’ and ‘learning’, corresponding adjectives were also included in the coding. This detailed scheme aims to systematically capture as many aspects and facets of the conceptual construct ‘digital resources’ as possible. Ultimately, 26 categories were developed and used to code all 23 articles.

Coding was performed by two members of the author team to determine interrater reliability. First, one article was coded collaboratively as an example to co-ordinate understanding, and discrepancies were resolved through discussion. All items were then double-coded to determine interrater reliability. Kappa was computed in addition to the percent agreement.

**Fig. 4:**
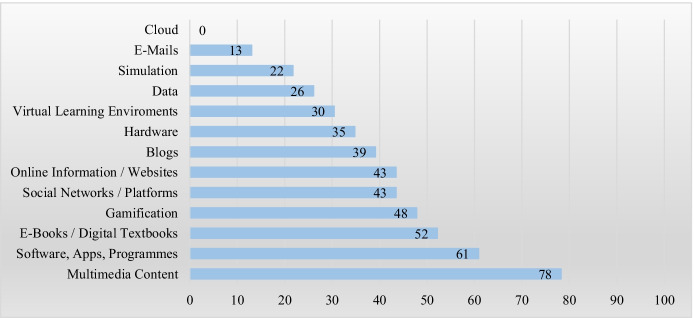
Percentage coding frequency

The individual codes were dichotomous (i.e., criterion is present/absent). The coders had to decide whether a criterion was met (1) or not (0). The kappa value was determined for each of the 26 categories. The mean overall kappa corresponds to κ = 0.857 with a standard deviation of 0.221. The kappa value for interrater reliability is very good, indicating reliable coding. The mean percentage agreement is 96.15% with a standard deviation of 4.32%. The ‘cloud’-category has the lowest match (κ = 0). The reason for this is that the base rate is 0 (see Fig. 4 in chapter 5.1). The category ‘Synonyms ➔ Technology’ reveals the next lowest match. The kappa value for this is κ = 0.465. Here, the problems of coding for the concept of digital resource become particularly clear. The term ‘technology’ is often either used inconsistently or it is unclear whether the term is a generic category of digital resources or a synonym for it. Given that the other categories have values from 0.7 to 1 and the maximum agreement of 1 was even achieved with 12 categories, the interrater reliability is also good to very good for the individual categories.

#### Existing definitions of digital resources

To provide a precise overview of the existing definitions, the relevant passages were first located, with a focus on signal words, including ‘define’, ‘definition’, ‘terminology’ or phrases such as ‘we mean…’, ‘refer to…’ or ‘we characterise…’. We also sought positions in which the term ‘digital resource’ was directly followed by examples. These were often explicitly referred to as examples (‘for example’, ‘e.g.’) and listed in brackets. The passages were compiled in a separate document to ensure categorisation independent of the rest of the context.

Analysis of the passages led to the classification of the existing definitions into three categories. Depending on the category, a deeper understanding of the concept of digital resources was achieved. Higher categories also met the criteria of those below. The categorisation is not an evaluation of quality but rather is intended as an overview of the existing definitions.

Category 1 – “basic” – includes all definitions that limit the understanding of the term ‘digital resource’ by simply naming examples. Category 2 – ‘advanced’ – includes definitions in which the examples are supplemented with descriptions of the term. In category 3 – ‘comprehensive’ – definitions are classified that, in addition to examples and descriptions, also include concrete distinctions from similar terms. Examples for the various categories are omitted here to prevent duplication of results.

Interrater reliability was also determined for the definitions’ categorisation. The coding procedure was analogous to the coding of the aspects and facets using the document, which only contained the relevant text passages. The coders determined whether the category was existent (1) or not (0). Kappa was determined for each of the three categories. The mean overall kappa corresponds to κ = 0.883. with a standard deviation of 0.101. The kappa value for interrater reliability is thus very good. The mean percentage agreement is 97.10% with a standard deviation of 2.51%. The categories ‘basic’ and ‘advanced’ each recorded only one different coding. Finally, the third category included no deviation.

## Results

To better contextualise the results, we divided the presentation into three parts in accordance with our RQs. The absolute and relative frequencies resulting from the articles’ coding are used to provide descriptive statistics. This also reveals which aspects and facets the conceptual construct ‘digital resource’ includes in current educational research.

The ‘Qualitative findings’ section extends the definitions presented by the review articles, revealing the definitions for the conceptual construct ‘digital resources’ in current educational research. The definitions have also been classified.

The final section concerns the conceptual placement of knowledge about digital resources in the TPACK model. Since the localisation is closely linked to the definition of digital resources, this also represents a result of the review and is therefore part of the presentation.

### Descriptive statistics from coding

On first reading, the differences in content are noticeable. However, this concerns not small deviations in understanding but contrary views. While Ukah (2020) considers ‘digital resources’ a generic term that includes hardware and software, Ricardo-Barreto et al. (2020) considers digital resources an independent category that explicitly excludes hardware and software.

Numerous gradations exist between these two ‘extremes’, necessitating systematic coding of the articles in terms of the respective understandings of the term. Figure 4 depicts the frequency percentage of the various aspects and facets based on the total number of articles in this review.

More than 75% of the articles classify multimedia content (including videos, films, podcasts, graphics, music) as digital resources. 52% of the articles (12) count ‘e-books/digital textbooks’ among digital resources. Using the example of collaborative digital textbooks, Kempe and Grönlund (2019) showed that they encourage interaction—another key characteristic of digital resources.

However, coding of the digital resources’ aspects and facets as mentioned in the articles revealed that these are often closely linked to the focal points of the respective investigation. In their systematic review, Hehir et al. (2021) analyse the possibility of connection among students during the COVID-19 pandemic, counting emails among digital resources, similar to Navarro-Pablo et al. (2019), as they serve as a means of communication and feedback provision (Hehir et al., 2021). The teachers in Khoza's study regard emails not only as an opportunity for communication but also as a resource and basis for assessment (2018). One reason that other authors exclude emails from digital resources is offered by Rodríguez-Muñiz et al. (2021), who assert that email and Facebook are platforms via which digital resources can be delivered and made available to students. This reveals quite different views on the same medium regarding the notion of digital resources.

This possibility of providing and exchanging information makes platforms and social networks a digital resource for many authors, with 43% (10 articles) counting them among digital resources. The argumentation for this classification may be partially associated with Adler’s understanding of the term: ‘It is possible to think about resource as the verb re-source, to source again or differently’ (Adler, 2000, p. 207). Hence, Twitter and Facebook are ‘resources for exchange’ and thus starting points for new teaching ideas (Trouche et al., 2020).

Social networks and platforms are often sources of information also, which means that the argumentation can be transferred to the categories ‘blogs’ (39%; 9 articles) and ‘online information/websites’ (43%; 10 articles). In Maher et al.’s (2012) study, 45% of the surveyed teachers reported using ‘informational web pages that you locate before the lesson as a source of information’ ‘frequently’ and 41% ‘occasionally’ (p. 145). The use of digital resources may highlight the aim of teacher–student or student–student interaction. For this reason, Littlejohn et al. (2008) consider blogs (and wikis) ‘dynamic resources’ that ‘allow interaction and contribution’ (p. 762).

Our coding reveals the importance of digital resources in interaction processes in schools and classrooms since ‘gamification’ elements are part of the digital resources for 48% of the articles. As a digital resource, online games can serve different purposes through the possibility of interaction. For example, 77.1% of the student teachers surveyed by Gomez-Gomez (2021) stated that they participated in online games ‘to review the subject’ (p. 597).

The aspects and facets of the digital resources reported hitherto reveal their versatility. This is one of the reasons that no uniform understanding exists, accompanied with the wide range of applications dealing with digital resources. It is thus crucial to understand the applications themselves and their categorisation and assignment. With programmes, the focus is shifting from content delivery to content creation. Khoza (2018) specifically cites Microsoft Office. Navarro-Pablo et al. (2019) describe software, which they also count as digital resources, as follows: ‘Software (programmes for creating, running, managing and editing content)’. Altuna and Lareki (2015) do this similarly. However, Ricardo-Barreto et al. (2020) do not count programmes as a subcategory of software. Here, the aspects of delivery and creation make software a tool rather than a digital resource itself—a view that, as our coding revealed, is shared by several authors (e.g., Harley, 2007; Luetkemeyer & Mardis, 2016). Fourteen articles (61%) count the ‘Software, Apps, Programmes’ category among digital resources (61%).

The term ‘resource’ is not used consistently. Some articles appear not to distinguish the words ‘tool’ and ‘resource’, with nine using ‘tool’ interchangeably with ‘resource’. Thus, the word is the most frequently used synonym for ‘resource’ in this review. The same applies to ‘online’ as a synonym for ‘digital’ (9 articles, 39%). Fifteen articles emphasised the educational context with additions such as ‘learning’, ‘instructional’ or ‘educational’. It was not possible to distinguish whether these were mere rhetorical variations. However, this makes it difficult to define the term.

Overall, our coding and analysis of the articles reveals that focal points and perspectives must be accounted for to understand the individual authors’ use of the term ‘digital resources’. Even if only 30% of the articles (7) regard the ‘virtual learning environment’ as a digital resource, this assignment certainly makes sense from the learners’ perspective. The same applies to cloud services (0%) which, if used collaboratively, can facilitate exchange, like social networks and platforms. Hardware was regarded as a digital resource as a presentation or access medium by 35% (8), and data (26%, 6) and simulations (22%, 5) for certain subjects can be consulted as sources of knowledge. These abundant possibilities highlight the importance of digital resources for school and teaching.

### Qualitative results

In addition to the content differences, the form and detail of the definitions between the various articles also differ considerably. Ukah (2020) selected a simple enumeration of examples to define the term. Ricardo-Barreto et al. (2020) use ICT as a generic term and list hardware, software and digital resources as subcategories. For each subcategory, they explain in great detail what they mean by the respective term. Qualitative analysis of the definitions identified three categories into which the different representations within the articles can be classified (Table 3).

**Table 3:** Categorisation of articles

Level of understanding	Key aspects—hierarchical, i.e., higher levels include aspects of previous level(s)	Publications
3 – comprehensive	Concrete distinctions from similar terms	Ricardo-Barreto et al., 2020; Trouche et al., 2020
2 – advanced	Examples supplemented with descriptions	Altuna & Lareki, 2015; Ettazarini, 2017; Kervin et al., 2019; Khoza, 2018; Littlejohn et al., 2008; Maher et al., 2012; Pepin et al., 2017a, b; Remillard et al., 2021; Štemberger & Konrad, 2021; Wood et al., 2021
1 – basic	Naming examples	Cusi et al., 2017; Gomez-Gomez, 2021; Harley, 2007; Hehir et al., 2021; Kempe & Grönlund, 2019; Khine, 2006; Luetkemeyer & Mardis, 2016; Maiier & Koval, 2021; Navarro-Pablo et al., 2019; Rodríguez-Muñiz et al., 2021; Ukah, 2020

Ukah’s (2020) is assigned to ‘basic’ because it only enumerates examples in brackets. Nevertheless, the five examples reveal that the term ‘digital resource’ includes hardware, software and multimedia content (e-journals). The number of examples given ranges from three (Maiier & Koval, 2021) to sixteen (Harley, 2007). The latter can generate a more differentiated understanding of the term. Neither Maiier and Koval (2021) nor Harley (2007) consider hardware part of digital resources.

The variation is not only limited to the number but also affects the range of examples listed. Khine (2006) lists the following digital resources in his article: ‘PowerPoint presentations, video clips, animation, digital photographs and word documents’ (p. 130). In our coding scheme (see Appendix Table 4), these can all be assigned to the category ‘(multi-)media content’. Meanwhile, Gomez-Gomez’s (2021) examples open up the possibility for greater interactivity: ‘forums, online games, videos for virtual role-playing games, blogs and digital portfolios’ (p. 591). While Khine's (2006) focus is on the level of reception, Gomez-Gomez (2021) expands this to include communicative and interactive aspects

The ‘advanced’-category includes Altuna and Lareki’s (2015) definition. Here, digital resources are divided into seven different groups. Some groups also include examples and provide a better understanding. The group ‘instructional software’ is also annotated with an extra explanation. Thereby, digital resources are independent of the medium of (re)presentation. Instead, Maher et al. (2012) see digital resources in conjunction with an interactive whiteboard every time. Thus, the scope of application is determined and the explanation offers a better understanding of the term.

To belong to the ‘comprehensive’ category, there must be a concrete distinction from similar terms. This applies to only two articles in this review. One is that by Ricardo-Barreto et al. (2020). Here, digital resources are distinguished from hardware and software. All three categories include examples and explanations of the characteristics. This differentiation enables a relative precise idea of the understanding of the term. ‘Relative precise’ because at one point there is a deviation from the otherwise consistent use of the term. There, objects previously assigned to the hardware category are once referred to as resources (p. 412):

‘On the other hand, results show that some resources that require a greater infrastructure and/or additional investment, such as Clickers, digital board, and laser pointer, are less frequent elements.’

Even though this may be a sophistry on our part, this inconsequence can sometimes be very confusing. This applies, for example, to the article by Trouche et al. (2020). The article also strives for a distinction between similar terms. In contrast to Ricardo-Barreto et al. (2020), however, ‘digital technologies’ and ‘digital resources’ are distinguished. While a distinction is made between hardware and software in the ‘digital technologies’ category, digital resources are divided into OER and digital curriculum resources (DCR) (Trouche et al., 2020). At the latest, when the resource systems are presented the various distinctions are no longer considered. At this stage, everything is summarised under the term ‘digital resources’.

Hence, there are also qualitative gradations resulting from the different consistency in the use of the terms. It is thus evident that the definitions within and outside of their categories do not provide a uniform understanding of the digital resource as a concept.

### Conceptual placement in the TPACK model

The quantitative and qualitative results presented allow the location of the digital resources in the TPACK model. The authors’ various understandings of the terms can be traced back to different focal points, which can be divided into two groups: ‘knowledge about digital resources’ and ‘dealing with digital resources’. Although the latter does not explicitly contain knowledge as a concept, this group also presupposes knowledge. Based on the TPACK model, the two groups can be classified as follows:

‘Knowledge about digital resources’ includes above all technological knowledge (TK). In relation to the DigCompEdu, this can be seen in ‘Creating and Modifying’. Navarro-Pablo et al. (2019) classify ‘software’ as a digital resource and more specifically ‘programmes for creating, running, managing and editing content’ (S. 3). They thus refer to teachers’ knowledge of various programmes and their suitability for specific teaching purposes. At the same time, teachers’ knowledge about programmes is important for ‘selection’. Different file formats or system compatibility should also be considered when teachers select digital resources for their teaching. Maiier and Koval (2021) refer to the DigCompEdu, in which ‘digital files’ are explicitly mentioned as an example of digital resources (Redecker, 2017). However, for ‘sharing’, knowledge of CC licenses and copyright is key (Redecker, 2017). Beyond that, ‘sharing’ is closely related to ‘collaboration’ as one of the 4Cs (creativity, critical thinking, communication, collaboration (Bedir, 2019)) and OER. Trouche et al. (2020) illustrate this using the example of the teacher Anna and clarify that OER can also promote collaboration across institutional borders. Apart from collaboration, which is a competence, the other examples contain knowledge in the form of (pure) TK, with pedagogical or content aspects omitted for now.

This partly contradicts Mishra and Koehler, who consider that TK training alone is insufficient (2006). However, PCK is irrelevant to CC licences, which are context-independent. Nevertheless, it is important for collaboration, as the above example demonstrates. TK will also be increasingly important in connection with the so-called lock-in effect, which is becoming increasingly common in schools (Haller, 2018). TK training can ensure more targeted promotion of the corresponding competences.

‘Dealing with digital resources’ can also be assigned to TK. However, the focus shifts to the borders of neighbouring fields of knowledge. In addition to TK, CK and/or PK is also required. For example, the preconditions of the learning group must be considered when ‘modifying’ digital resources (Redecker, 2017). In this conjunction, it is a matter of PK that teachers acquire through interaction with their students. In turn, the ‘modifying’ itself requires technical knowledge and concerns—depending on the focus—the transition from TK to TPK. On the other side is CK. Various digital resources that serve to visualise, supplement or even replace (e.g., experiments) can be used for different subjects. CK and understanding of the transfer from real to simulation experiment is particularly helpful in integrating simulations. For example, Aktaş and Özmen (2022) introduced the TPACK model as part of a course for prospective teachers and gave an introduction to PHET, ‘an interactive and research-based science simulation’ (p. 9). The aim of this introduction was ‘to gain the ability to use ICT tools’ (p. 9). The participants waited until then to begin planning the lessons. This means that the knowledge of ‘dealing with digital resources’ (here: simulations) is at the intersection between TK and TCK because these are subject-specific simulations. If the simulation’s didactic use in the classroom is included in the planning, a teacher must consider knowledge from all three components, as expressed in the model through the TPACK-juncture.

**Fig. 5:**
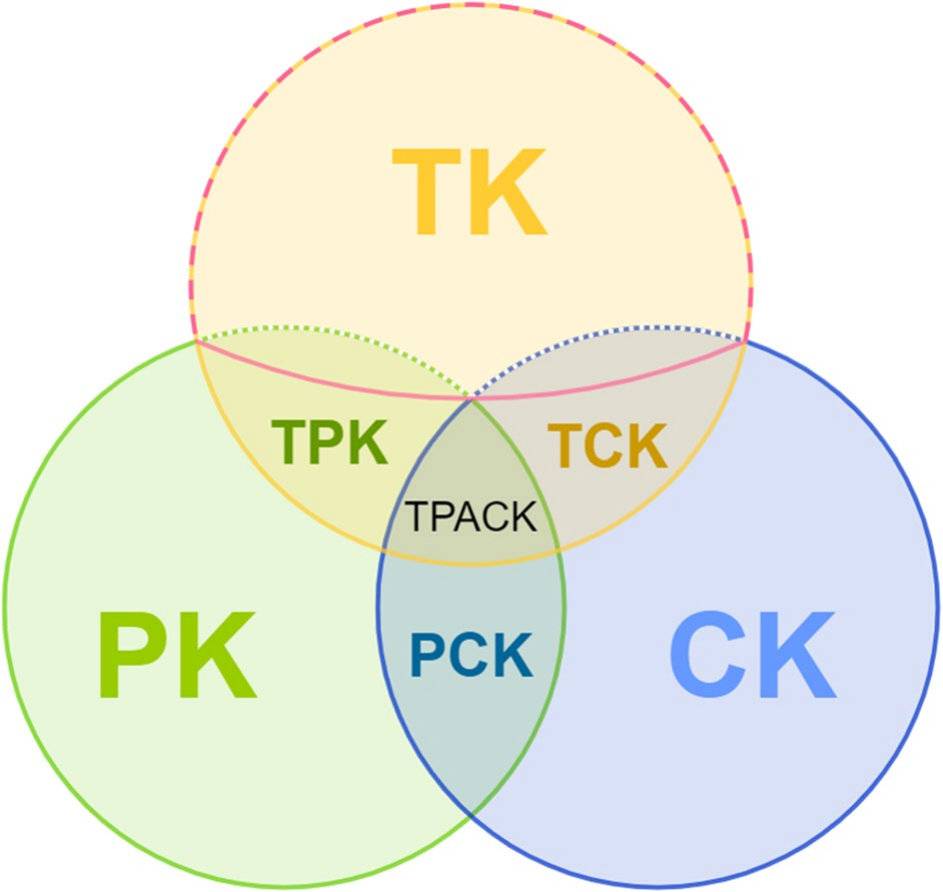
Indicating Digital Resources in the TPACK model technological knowledge (TK), pedagogical knowledge (PK), content knowledge (CK), technological pedagogical knowledge (TPK), technological content knowledge (TCK), pedagogical content knowledge (PCK), technological pedagogical content knowledge (TPACK)

In summary, ‘knowledge about digital resources’ may be regarded as (pure) TK. However, depending on the selected or necessary focus, ‘dealing with digital resources’ also includes PK and CK and thus affects the intersection with TPK, TCK and TPACK. Figure 5 illustrates in red the area in which the digital resources can be located. The knowledge components involved, which play a role in the competence area of digital resources, once again reveal the complexity and importance of this field. Both must be considered in teachers’ training and further education. Beyond that, this differentiated view also influences the definition of digital resources and must be included in it. This is the only means of promoting digital resources competences in a comprehensive and targeted manner.

## Discussion

**Fig. 6:**
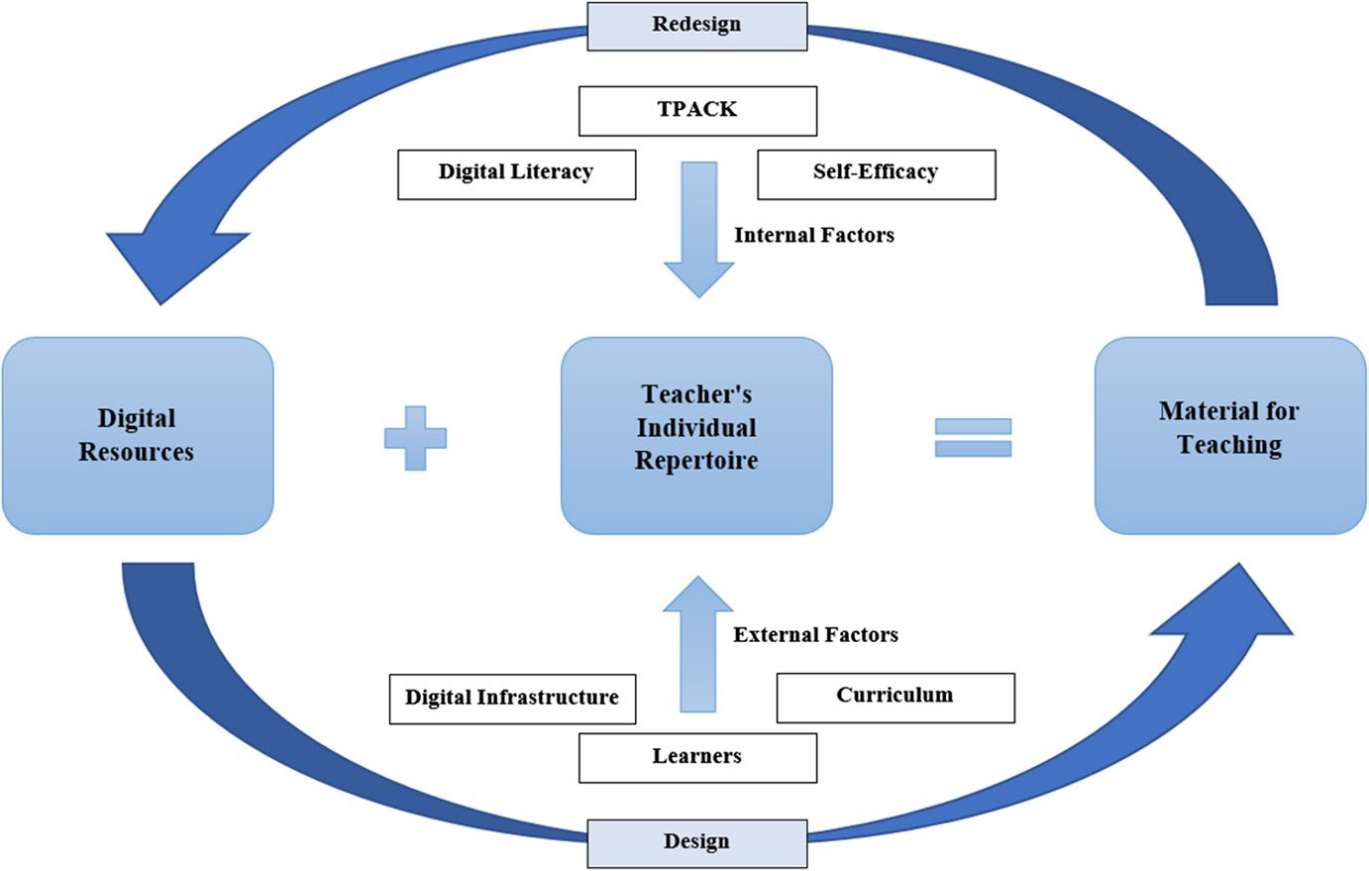
Designing digital resources

Digital resources—understanding them, knowing about them and teachers’ professional competence in dealing with them—are complex (Kervin et al., 2019; Khoza, 2018; Štemberger & Konrad, 2021). New challenges for (prospective) teachers that Koehler and Mishra (2009) addressed and that were further explored by Mußmann et al. (2021) 12 years later are also clearly recognisable in the area of digital resources. Teachers’ important role (not only) in connection with digital resources was repeatedly emphasised (Cusi et al., 2017; Štemberger & Konrad, 2021). For this reason, the importance of teacher educators is also highlighted (Ungar & Baruch, 2016). Even if ‘knowledge about digital resources’ can be viewed in isolation (OER, CC licences, etc.), only ‘dealing with digital resources’ facilitates innovation (Napal et al., 2020). Innovation through interaction is probably the most concise summary of the results in this review. Digital resources themselves do not bring innovation. Instead, the pedagogy and thus the interaction of teachers and students must also experience innovation (Bottino, 2020). To describe the associated chain of thought and the integration of digital resources in teaching and learning, we recommend an action-theoretical model— ‘designing digital resources’ (Fig. 6) —facing the fact that the design-process ‘plays a crucial role to take advantage of digital resources’ (Lopes & Costa, 2019). This schematic representation should also facilitate recognition and understanding of the complexity described above.

The model was developed following the ‘documentational approach to didactics (DAD)’ (Trouche et al., 2018) but transcends the mathematical context and specifically concerns digital resources. The interaction process was examined more specifically, and the various influencing factors are linked to the ‘competence as continuum’ model by Blömeke et al. (2015).

The DAD assumes that due to teachers’ individual schemes of usage, different documents are created from the same resources (resource + scheme of usage = document) (Trouche et al., 2018). If this is transferred to digital resources, different teachers develop different materials from or with the same digital resources (Bottino, 2020). The distinction between ‘from’ and ‘with’ once again refers to the results of this review. ‘From digital resources’ concerns, for example, OER or multimedia content that teachers have as a starting point for a possible adaptation. ‘With digital resources’ refers to hardware, software and programmes necessary for creation. Furthermore, the term ‘material’ is used explicitly. Since digital resources facilitate more flexible handling (Ricardo-Barreto et al., 2020), ‘material for teaching’ denotes not only texts but also multimedia content of every description as well as apps and simulations. ‘Material for teaching’ is simultaneously an expression of the teacher’s performance. In their competence model, Blömeke et al. (2015) considered performance as part of the continuum on which competence is located. By incorporating the various factors detailed below into the material design process, the teacher shapes the material (Kempe & Grönlund, 2019). If further adaptations must be made after using the material in the classroom the teacher must revise the material.

This process is illustrated by the return arrow. The development process is not linear but runs cyclically or spirally. Similar to the design-based research (DBR) approach, whose rate of use in the education sector rapidly increased in the last period (Dağhan & Gündüz, 2022), material developed from digital resources can again be provided as a new digital resource. The processes are named ‘design’ and ‘re-design’ accordingly. Teachers thus act as designers (Kempe & Grönlund, 2019; Khine, 2006). Teachers can also be viewed as users of existing digital resources. By changing and sharing them, they become ‘content providers’ (Kempe & Grönlund, 2019). To vary digital resources, the teacher resorts to their individual repertoire. We deliberately avoid the term ‘scheme of usage’ and follow the definition of Mishra and Koehler (2006), who cite Wasley et al. (1997): ‘[R]epertoire is defined as “a variety of techniques, skills, and approaches in all dimensions of education that teachers have at their fingertips”’. Thus, the individual repertoire is influenced by various factors.

Internal factors are instrumental, in particular, the attributes each respective teacher brings due to their own competences and experience. In this context, Lachner et al. (2019) speak of teachers’ conceptual knowledge. This is mainly influenced before and during university education and changes only slightly for in-service teachers (König et al., 2020; Lachner et al., 2019). According to Blömeke et al.’s (2015) model, this is ‘disposition’. It thus includes ‘knowledge about digital resources’ (TK) and, as a connecting element to the external factors, ‘dealing with digital resources’ (TPK, TCK, TPACK), as well as the remaining knowledge elements. Competences in the sense of ‘digital literacy’ also play an important role here (Yurinova et al., 2022). However, several studies proved that the deployment and use of digital resources is largely determined by teachers’ self-efficacy (Luetkemeyer & Mardis, 2016; Maiier & Koval, 2021). Hence, this is representative of the more comprehensive area of ‘affect motivation’.

The external factors, which contribute to the development of situational knowledge (Lachner et al., 2019), must also be considered. These are comparable to the ‘situation-specific skills’ (König et al., 2021a; Blömeke et al., 2015). For selection, creation and modification, the teacher must monitor the current teaching situation and the usability of digital resources (Estrada-Molina et al., 2022). In addition to the curriculum and learning group requirements, this includes the digital infrastructure. It is not only in Germany that teachers must deal with the lack of digital equipment in schools and adapt their lesson planning accordingly (Ricardo-Barreto et al., 2020). Luetkemeyer and Mardis (2016) noted that ‘bandwidth also emerged as a necessary conveyance of video, large data sets, simulations, and other resource types that are best used over high-speed Internet connections’ (p. 13).

Interaction is crucial for all aspects and processes of the model (Kervin et al., 2019). Hehir et al. (2021) showed that ‘connectedness’ plays a crucial role in the learning process. During the pandemic, this was made facilitated by digital resources only (Hehir et al., 2021). In addition, digital resources only develop further through interaction.

Sandanayake et al. (2021) identified the different interactions in online learning that can be transferred to digital resources. Learners interact with digital resources, which may require the teacher to revise and adapt them (as mentioned at the beginning of this section). This affects the re-design process. Teachers also interact with digital resources and their adaptations as well as their presentation and purposeful use in the classroom have a major impact on the students’ learning process (Willis et al., 2019). The interaction between the students and between teachers and learners in turn affects the teacher’s individual repertoire (Menninga et al., 2021) and thus the design process, revealing the close interdependencies and how the individual threads converge with the teacher. The SAMR model’s attempt to innovate the teaching–learning process through digital technologies (i.e., digital resources) is misguided (Eickelmann, 2011; OECD, 2015). As Kervin et al. (2019) stated in their principles 1 & 2, ‘digital resources cannot replace the work of a teacher’ and ‘no application can replace human interactions with the teacher’ (p. 11). Innovation is made possible by the interaction of all involved and the digital resources.

## Limitations

The articles in this review did not explicitly try to define the conceptual construct of ‘digital resources’ or place greater value on consistent use. Similar terms are often poorly distinguished, and distinct coding was not always possible. The definition resulting from the synthesis of the different uses also represents an attempt to compensate the range of meaning.

Since the selected articles’ relevance was initially assessed based only on the abstracts, it is also possible that some definitions of the conceptual construct that appear in the full-texts were not considered here.

## Digital resources

The review revealed the importance of digital resources in education and the great contribution that teachers make, assuming different roles in dealing with digital resources (Bottino, 2020). The many different aspects and facets of digital resources cannot be expressed concisely. For this reason, perspective (e.g. students, teachers) and focus (e.g. scientific, linguistic, etc.) must always be considered when defining digital resources.

Digital resources in education have a digital or technical aspect and serve as a source of knowledge, innovation, and interaction. They are created and accessible on hardware, such as computers, smartphones, laptops, and interactive whiteboards (Estrada-Molina et al., 2022). Therefore, we count hardware as a digital resource because, used as a presentation medium, it also serves as a source of knowledge.

As the review has shown, the definition is also determined by the use and application of digital resources. Thus, we distinguish between digital resources for content delivery and for content creation. Content delivery includes communication and connection between the different actors in the school context and the provision and exchange of feedback and information. Digital resources in this area include, for example, emails, digital textbooks, social networks, platforms, blogs, clouds, websites, online information, and virtual learning environments. Here the focus is on interaction. Content creation primarily refers to the development, modification, and design of teaching materials. For example, software, apps, programmes, and simulations are considered digital resources. Innovation plays an important role here. The teacher can influence the teaching–learning process by using his or her didactic skills and by taking the current situation into account, thus contributing to its innovation. Above all, there is multimedia content. As a digital resource, it serves as the starting point for both creation and delivery. It is the foundation for innovation, interaction, and knowledge in twenty-first century schools. This is reflected by the topical foci chosen by the articles of this review (Fig. 4).

## Future research

This review’s findings should be incorporated into future educational research. Above all, they must be considered for the targeted promotion of (prospective) teachers’ digital competences. With this in mind, we intend to develop a suitable competency test for knowledge that serves to analyse how pre-service and in-service teachers may be supported in promoting their skills to master the complex work and enable competent digital resource use. To reach those goals, empirical research is needed to understand the link between associated teacher skills and teacher performance as outlined in designing digital resources (Fig. 6). Assumptions underlying our model in Fig. 6 needs rigorous measurement of associated teacher skills and performance as well as in-depth empirical examination in future research.

## Data Availability

Not applicable.

## Appendix

Table 4

**Table 4:** Coding scheme with dichotomous coding (criterion is present = 1, criterion is not present = 0)

	Coding category	Sample paper 1 (Cusi et al., 2017)	Sample paper 2 (Navarro-Pablo et al., 2019)
Based on DigCompEdu	Hardware (PC, Laptop, Smartphone, Tablet, Smart-/Whiteboard)	1	1
	Software, Apps, Programmes	1	1
	Social Networks /Platforms	0	1
	Online Information, Websites	0	1
	Multimedia content (Images, Audio, Video)	0	1
	Virtual Learning Environments	0	1
	Gamification (Game, Test, Quiz)	0	1
	Data (Need to be analysed, treated and/or interpreted)	0	1
Extension to DigCompEdu	Blogs	0	1
	E-Mails	0	1
	E-Books/ Digital Textbooks	1	1
	Simulation	0	0
	Cloud	0	0
	Concrete Example	0	0
Synonymous to Resource	Device	0	0
	Tool	0	1
	Artefact	0	0
	Technology	0	1
	ICT	0	0
Synonymous to Digital	Multimedia	0	0
	Technological	0	0
	Electronic	0	0
	ICT	0	0
	Online	0	1
	Supplement (learning/ instructional/ educational/ curriculum)	0	0
	Related to DigCompEdu	0	0
